# “Anxiety is not cute” analysis of twitter users’ discourses on romanticizing mental illness

**DOI:** 10.1186/s12888-024-05663-w

**Published:** 2024-03-21

**Authors:** Barikisu Issaka, Ebenezer Ato Kwamena Aidoo, Sandra Freda Wood, Fatima Mohammed

**Affiliations:** 1 Department of Advertising and Public Relations, Michigan State University, East Lansing, USA; 2 Communication Studies Department, University of Iowa, Iowa City, USA; 3 Hugh Downs School of Human Communication, Arizona State University, Tempe, USA; 4 Department of Information Systems , University of Nevada, Reno, USA Reno,; 5 Michigan State University, Lansing, USA

**Keywords:** Social media, Mental health, Romanticization, Discourse, Twitter, Authentic discussions, Destigmatization, Topic modeling, Responsible discussions

## Abstract

**Background:**

The proliferation of social media platforms has provided a unique space for discourse on mental health, originally intended to destigmatize mental illness. However, recent discourses on these platforms have shown a concerning shift towards the romanticization of mental health issues. This research focuses on Twitter (now called X) users’ authentic discussions on the phenomenon of romanticizing mental health, aiming to uncover unique perspectives, themes, and language used by users when engaging with this complex topic.

**Methods:**

A comprehensive content analysis was conducted on 600 relevant tweets, with the application of topic modeling techniques. This methodology allowed for the identification and exploration of six primary themes that emerged from Twitter users’ discussions. Statistical tests were not applied in this qualitative analysis.

**Results:**

The study identified six primary themes resulting from Twitter users’ discussions on the romanticization of mental health. These themes include rejecting/critiquing the glamorization of mental health, monetization of mental health by corporate organizations, societal misconceptions of mental health, the role of traditional media and social media, unfiltered realities of depression, and the emphasis on not romanticizing mental health.

**Conclusions:**

This study provides valuable insights into the multifaceted discourses surrounding the romanticization of mental health on Twitter. It highlights users’ critiques, concerns, and calls for change, emphasizing the potential harm caused by romanticizing mental illness. The findings underscore the importance of fostering responsible and empathetic discussions about mental health on social media platforms. By examining how Twitter users interact with and respond to the romanticization of mental health, this research advances our understanding of emerging perspectives on mental health issues among social media users, particularly young adolescents. The study also underscores the effects of this phenomenon on individuals, society, and the mental health community. Overall, this research emphasizes the need for more responsible and knowledgeable discussions around mental health in the digital age.

## Background

Mental illness (MI) is characterized by significant disturbances in thoughts and emotions [1]. The World Health Organization (WHO) reports that approximately one billion individuals, equating to over one-eighth of the global population, grapple with mental disorders, with depression impacting 280 million and anxiety affecting 301 million [2, 3]. Despite the prevalence of MI, persistent stigma surrounds it, often driven by historical fears and media portrayals [4]. Corrigan et al. [5] identified two forms of stigma associated with mental illness: internalized stigma, where individuals within the stigmatized group develop negative self-perceptions; and public stigma, encompassing society’s adverse attitudes leading to prejudice and discrimination [5].

The emergence of social media has revolutionized the discourse on mental health, providing a platform for open discussions and education [6, 7]. Social media enables individuals to share their experiences, reducing stigma and initiating conversations [8]. However, social media has also given rise to a concerning trend—the romanticization of mental illness, portraying it as glamorous and desirable [9–11]. Some users normalize mental illness by treating it as an accessory and characterizing its challenges as a form of victimization [12]. This phenomenon distorts the true nature of mental illness and may encourage it as an alternative form of self-expression [4]. While prior research has explored romanticization in traditional media [9, 12], a significant gap exists in understanding how social media users perceive and discuss this phenomenon on platforms like Twitter. Existing studies have predominantly examined content analysis and framing in traditional media, overlooking the unique perspectives and language employed by users in the social media context. This study aims to analyze how Twitter users discuss the issue of romanticizing mental health on the platform, addressing the gap in the literature and providing insights into this specific social media context. It seeks to inform policies and interventions addressing the challenges posed by mental health romanticization on Twitter.

### Mental health in traditional media

Traditional media, including movies, has a history of inaccurately portraying mental illnesses, perpetuating negative stereotypes across various cultural contexts. Wood et al. [13] observed that mental illness has frequently featured in mass media, but these representations have consistently fallen short. They tend to depict mental illnesses as violent, unpredictable, overly simplistic, detached from reality, or innocent [14].

These portrayals often serve as narrative devices, aiding in character development and scene establishment [15]. By presenting mentally ill individuals as behaving and appearing distinct from “normal” individuals, these depictions reinforce an “us vs. them” narrative [16]. Hyler and colleagues identified and examined six recurring movie clichés that further stigmatized individuals with mental illnesses, including the narcissistic parasite, seductress, enlightened member of society, rebellious free spirit, and homicidal lunatic [17]. Numerous studies conducted across diverse cultural contexts have highlighted the prevalence of these inaccurate representations of mental health in traditional media. For example, studies have shown that news coverage in various countries can link mental illness to violence [18]. News stories often attribute gun violence to “dangerous people “rather than “dangerous weapons,” perpetuating stigmatizing narratives [19].

Similarly, Corrigan et al. [5] reported that a substantial portion of newspaper stories in different regions portrayed mental illness as dangerous and violent, with such stories frequently featured prominently. Misrepresentations of mental illness are not limited to newspapers but extend to global literature, including novels. Works like “Liar” (2009) by Justine Larbalestier depict individuals with mental illness as criminal and dehumanized, potentially perpetuating inaccurate perceptions across international audiences [20]. Films like “All the Bright Places” illustrate how the stigma associated with mental illness exacerbates characters’ mental health challenges, impacting individuals from diverse cultural backgrounds within the narrative [21].

Apart from stigmatization, mental illnesses like OCD and ADHD are also frequently trivialized. One aspect of trivialization involves the normalization of symptoms associated with mental illness, which may result in the dismissal of these symptoms as commonplace experiences rather than indicators of significant distress or impairment. For example, behaviors related to obsessive-compulsive disorder (OCD), such as frequent handwashing or checking rituals, may be trivialized as mere quirks or personality traits rather than recognized as symptoms of a debilitating mental health condition. Additionally, trivialization can manifest through societal attitudes and media portrayals that depict mental illness in a lighthearted or humorous manner, thereby minimizing the lived experiences of individuals affected by these conditions. This can contribute to a lack of empathy and understanding toward those struggling with mental health challenges.

Research has shown that trivialization of mental illness can have detrimental effects on help-seeking behaviors and treatment adherence among individuals experiencing psychological distress. When symptoms are trivialized or dismissed, individuals may feel invalidated or reluctant to seek professional help, fearing that their concerns will not be taken seriously [68, 69]. Furthermore, trivialization intersects with other forms of stigma, such as social rejection and discrimination, exacerbating the challenges faced by individuals with mental health conditions in various social and institutional settings.

Several studies have contributed to our understanding of trivialization in relation to mental illness. For example, Clemente and colleagues [68] conducted qualitative research to understand meanings and implications of the stigma related to bipolar disorder and found that trivialization of their symptoms by others contributed to feelings of frustration and alienation, it further contributed to cause of treatment refusal. This demonstrates how trivialization represents a significant barrier to the recognition and support of individuals with mental health conditions in society.

The literature discussed above underscores the historical prevalence of unfavorable portrayals of mental health in traditional media, transcending national borders and encompassing various cultural contexts, including TV, movies, novels, and fictional characters. Providing a brief explanation of how mental health has been depicted on social media would offer valuable insights and enhance the contextual understanding of this study.

### Social media and mental health

In the past decade, social media has undergone a profound transformation, reshaping the way individuals interact, communicate, and address mental health concerns [22]. It has evolved into a dynamic platform for self-expression, enabling people to openly share their thoughts, life experiences, and memories. Moreover, it has become a virtual space where individuals grappling with mental health challenges can connect, find solace, and extend their support to like-minded peers [23]. Research has illuminated the prevalence of constructive and positive discussions surrounding mental health on social media. For instance, it’s worth noting that tweets conveying inspirational messages tend to receive more retweets (an average of 4.17) than stigmatizing content (with an average of 3.66), underscoring the pervasive presence of uplifting messages in the digital sphere [24]. The discourse encompassing mental illnesses like depression and anxiety on social media overwhelmingly leans toward supportiveness and encouragement, creating a sense of solidarity among individuals facing similar struggles [25]. Young people, particularly, utilize platforms such as.

Facebook and Twitter as digital sanctuaries to escape external pressures that can adversely affect their mental well-being [26]. In essence, social media platforms serve as open canvases for unfiltered expressions and candid discussions about mental health, cultivating a sense of online community [27, 28]. Studies reveal that discussions on social media related to depression primarily revolve around offering support and sharing personal experiences, rather than perpetuating stigma or shame [29]. Similarly, research conducted on Sina Weibo, a prominent microblogging platform in China, illustrates that influential users tend to generate more supportive and helpful posts than negative or stigmatizing ones [30].

Information concerning recovery and treatment plays a particularly pivotal role in nurturing a positive discourse on mental health. These findings not only underscore the essential role of social media in dismantling the stigma associated with mental health issues but also emphasize its capacity to foster a compassionate and supportive digital community. In this light, social media emerges as a potent tool for advancing mental health awareness and advocacy [19]. These findings emphasize the profound potential of social media to reshape how we approach mental health, offering not only a space for candid conversations but also a powerful means to combat stigma and promote understanding and support among individuals facing mental health challenges.

### Suicide contagion and responsible media reporting guidelines

The issue of suicide contagion, particularly in contexts where romanticization occurs, has garnered significant attention in academic literature due to its potentially dangerous implications. Gould and Shaffer [70] and subsequent studies have shown that media portrayals of suicide, particularly those glamorizing or sensationalizing the act, can lead to copycat suicides, especially among vulnerable populations like adolescents. Efforts to address this issue include guidelines from the World Health Organization (WHO), Samaritans, the American Foundation for Suicide Prevention, and other mental health and prevention organizations. These guidelines seeks to streamline and deglamorize media reporting of suicide in a responsible manner more specifically news and media information. Among these guidelines is the avoidance of glorification of suicide to help prevent the Werther effect and possible imitation or contagion effects [71, 72]. However, despite these guidelines, research by Stack [74] and others suggests that sensationalized or glamorized media reporting on suicide persists, contributing to increased suicide rates, as evidenced by studies such as Fu and Yip [75] in Asia.

In recent years, attention has turned to the role of social media platforms like Twitter in influencing suicidal behavior. While social media offers real-time engagement and information dissemination, it also presents challenges in monitoring and regulating content related to suicide. Research by Markman [76] and Jashinshy et al. [77] has shown associations between Twitter activity related to suicide and actual suicide rates, prompting platforms like Twitter to update their policies on harmful content [78]. However, the implementation and enforcement of these policies remain a concern, as studies suggest limited uptake of recommendations. Therefore, despite efforts to address suicide contagion through media guidelines and social media policies, further research and action are needed to effectively mitigate the risks associated with romanticized depictions of suicide in digital environments.

### Romanticizing mental health on social media

While discourses on social media were initially aimed at destigmatizing mental health, there has been a notable shift towards romanticizing mental illness, including the creation of memes and content glamorizing conditions such as anxiety [21]. Users, in their attempt to make conversations about mental illness empathetic, often inadvertently end up romanticizing it, portraying it as an accessory, and those experiencing it as martyrs [31]. This romanticization can manifest in various forms, from seemingly harmless expressions to more troubling instances such as the glorification of suicide [4]. Chen [32] shared her experience of encountering romanticized mental illness content online, highlighting how depression and anxiety were portrayed as fashionable. This phenomenon extends to the proliferation of melancholic content, black-and-white imagery, and narratives of low self-esteem on platforms like Tumblr.

Similar concerns are observed on platforms like Facebook and blogs, where mental health disorders, including anorexia nervosa, self-harm, depression, and anxiety, are increasingly romanticized [1]. For instance, pro-anorexia (pro-ana) websites have gained notoriety for promoting dangerous behaviors linked to anorexia nervosa, such as extreme dieting practices and unhealthy weight loss methods [79]. These platforms often perpetuate unrealistic body ideals and encourage individuals to pursue thinness at any cost, disregarding the severe physical and psychological consequences of eating disorders [80].

Pro-ana websites, for instance, promote dangerous behaviors related to anorexia, perpetuating unrealistic dieting tips and methods [33]. Furthermore, the influence of celebrities like Lana Del Rey, who has incorporated themes of depression into her music and videos, contributes to the normalization of mental health struggles [1]. This trend has serious consequences, including the normalization of suicidal behaviors among young people, a rise in feigned mental illness, and self-diagnosis [9, 31]. It distorts societal perceptions of mental health and hinders genuine expressions of mental health concerns [12].

However, the use of pro-ana communities extends beyond the promotion of harmful behaviors, as they also serve as complex online environments where individuals with eating disorders find social support and understanding. Research suggests that individuals who engage with pro-ana content may do so as a means of coping with the challenges of their disorder, seeking validation from peers who share similar struggles [80, 81, 82, 83]. Within these communities, individuals often engage in open and candid discussions about their experiences with disordered eating, sharing personal stories, struggles, and successes. For many, the sense of belonging and camaraderie found in these online spaces provides a form of support that may be lacking in their offline lives [72].

Moreover, some individuals perceive pro-ana websites as empowering spaces where they can exercise agency over their bodies and identities, challenging societal norms and expectations [73]. This perception underscores the complexity of motivations underlying engagement with pro-ana content and highlights the need for a nuanced understanding of these online communities. Despite the potential benefits of social support within pro-ana communities, it is essential to acknowledge the inherent risks associated with these platforms. Exposure to pro-ana content has been linked to increased body dissatisfaction, disordered eating behaviors, and decreased motivation to seek professional help [81, 84]. It is important to acknowledge that pro-ana websites represent multifaceted online environments where individuals with eating disorders navigate between the promotion of harmful behaviors and the search for social support and understanding.

Romanticization of mental illness can be viewed as a contemporary form of stigma, akin to historical media stereotypes [4]. While prior research has explored various aspects of this phenomenon, including its framing and negative effects, these studies have mostly focused on traditional media. There is a noticeable gap in understanding how social media users engage in discussions about and articulate the romanticization of mental health in their own words on the platform. Furthermore, while studies have examined this issue on other social media platforms, Twitter has received limited attention despite its significant role in mental health discourse [34, 35]. Accordingly, there is a critical need to directly explore authentic discourses initiated by Twitter users. Twitter users frequently adopt pseudonyms and tend to interact with strangers, which allows them to communicate anonymously. Analyzing discourse on Twitter could offer a less biased representation of people’s experiences, as it is more naturalistic, includes a diverse population, and is often anonymous, thereby mitigating some of the constraints of conventional data collection approaches [36].

## Research question

How do Twitter users discuss the phenomenon of romanticizing mental health in their own words on the platform, and what themes and perspectives emerge from their conversations?

This research question aims to uncover the unique perspectives and language employed by Twitter users as they actively engage with the topic of romanticizing mental health, providing an in-depth exploration of their authentic discussions and viewpoints. By focusing on Twitter, it addresses the existing gap in the literature, emphasizing the importance of understanding the specific dynamics of this platform in shaping conversations about mental health romanticization.

## Methodology

### Content analysis

Our research methodology involved conducting content analysis on Twitter data using topic modeling. Content analysis is a commonly employed qualitative research method [37] that allows for a comprehensive examination of individuals’ experiences by extracting relevant topics from textual documents [38].

### Research setting and ethics

Twitter, with 166 million active users worldwide [39], was the research platform chosen. It is a prominent social networking site (SNS) for sharing daily activities, expressing opinions, and delivering news [40]. This unique platform enables the investigation of health and illness narratives [41], traditionally studied through methods like interviews and autobiographies [42]. Unlike conventional human subject research, analyzing public tweets does not require informed consent because such tweets are public [43, 44, 45]. However, this pertains solely to public tweets, as private profiles are excluded. Adhering to ethical guidelines [45], the study omitted user-identifying information and respected the privacy of individuals with private profiles [36].

### Data collection

Researchers collected data from Twitter using the 4CAT, a reference and analysis tool developed by researchers at the University of Amsterdam [46]. We inductively identified words associated with romanticizing mental health through earlier research [4, 21]. This resulted in a list of specific keywords which were used to scrape tweets using the following query:

#Mental health month #Romanticizing mental health# Mental health aesthetics #mental health is not cute #Mental health #glamourizing mental health # Mental health in the US #Romanticizing mental health in the US

To ensure the inclusion of mental health-related tweets, data analysis focused on a specific period, spanning from May 1st, 2022, to May 30th, 2023, corresponding to the global mental health month [47]. Researchers retrieved 1000 tweets. The observations gathered for analysis included key information such as username, followers, following, tweet ID, text, date, and time tweeted [36]. To maintain relevance, only tweets that specifically addressed the topic of mental health and romanticization were included, while tweets with irrelevant content or media attachments were excluded [48]. The final dataset was limited to English-language tweets, and after removing spam and public service announcements, a comprehensive set of relevant tweets (600 in number) was obtained.

### Data analysis

Latent Dirichlet Allocation (LDA) was employed as the topic modeling algorithm in this study. The primary goal was to discover latent topics within the text corpus and examine the distribution of topics across the documents. The model used is a powerful technique for extracting hidden themes or topics from a collection of text documents which is Latent Dirichlet Allocation (LDA). LDA was selected due to its ability to uncover hidden themes or topics within a corpus of text. This makes it a valuable tool for exploring various types of text data, such as customer reviews and news articles.

LDA is a hierarchical Bayesian model with three layers that is sought to investigate latent schemes in document corpora. Each document is represented as a random mixture of subjects, with each topic characterized by a distribution across words [49]. LDA is built on top of the premise that each document can be described by the probabilistic distribution of topics, and each topic can be described by the probabilistic distribution of words. This can provide a much clearer vision of how topics are connected [50].

Prior to applying LDA, the text data underwent a series of preprocessing steps such as tokenization, bag-of-words. The preprocessing is aimed to enhance the modeling accuracy and avoid the influence of search-related terms on the discovered topics. The model was trained using an optimum number of 5 topics. To determine the optimal number of topics (K), we used coherence and perplexity scores.

We experimented with different values of K, ranging from 2 to 11, and evaluated the coherence and perplexity interpretability of the resulting topics. A moderate range, such as 2 to 11 was chosen because training LDA with a very large number of topics may not lead to meaningful insights. If the number of topics selected is too small, the meaning under each topic will be too broad; if the number of topics selected is too large, it will lead to an over-clustering of data, producing useless topics or topics with too much similarity [51]. It also facilitates basic understanding of the topic’s structure. Seeing the same key words being repeated in multiple topics is a sign that ‘k’ is too large. Topic coherence can be defined as how interpretable a topic is based on the degree of relevance between the words within the topic itself [52]. Higher coherence scores indicate better-defined topics.

Perplexity is a measure of how well our LDA model predicts the held-out or unseen data. It quantifies how surprised the model is when it encounters new documents. Lower perplexity scores indicate that the model is better at predicting unseen text. Basically, the lower the perplexity, the more predictable it is. This indicates better generalization and performance [53]. Having the right balance between quantitative metrics like perplexity and qualitative assessments of topic quality is key to a successful LDA analysis. After careful consideration of the coherence perplexity scores, an optimal number of 5 topics was selected. The 5 Topics have a coherence score of 0.43 and perplexity score of -6.077. To finish with, we generated word clouds for each category of topic to visually represent the most important terms in each topic, which will be discussed in the analysis.

## Results

**Fig. 1:**
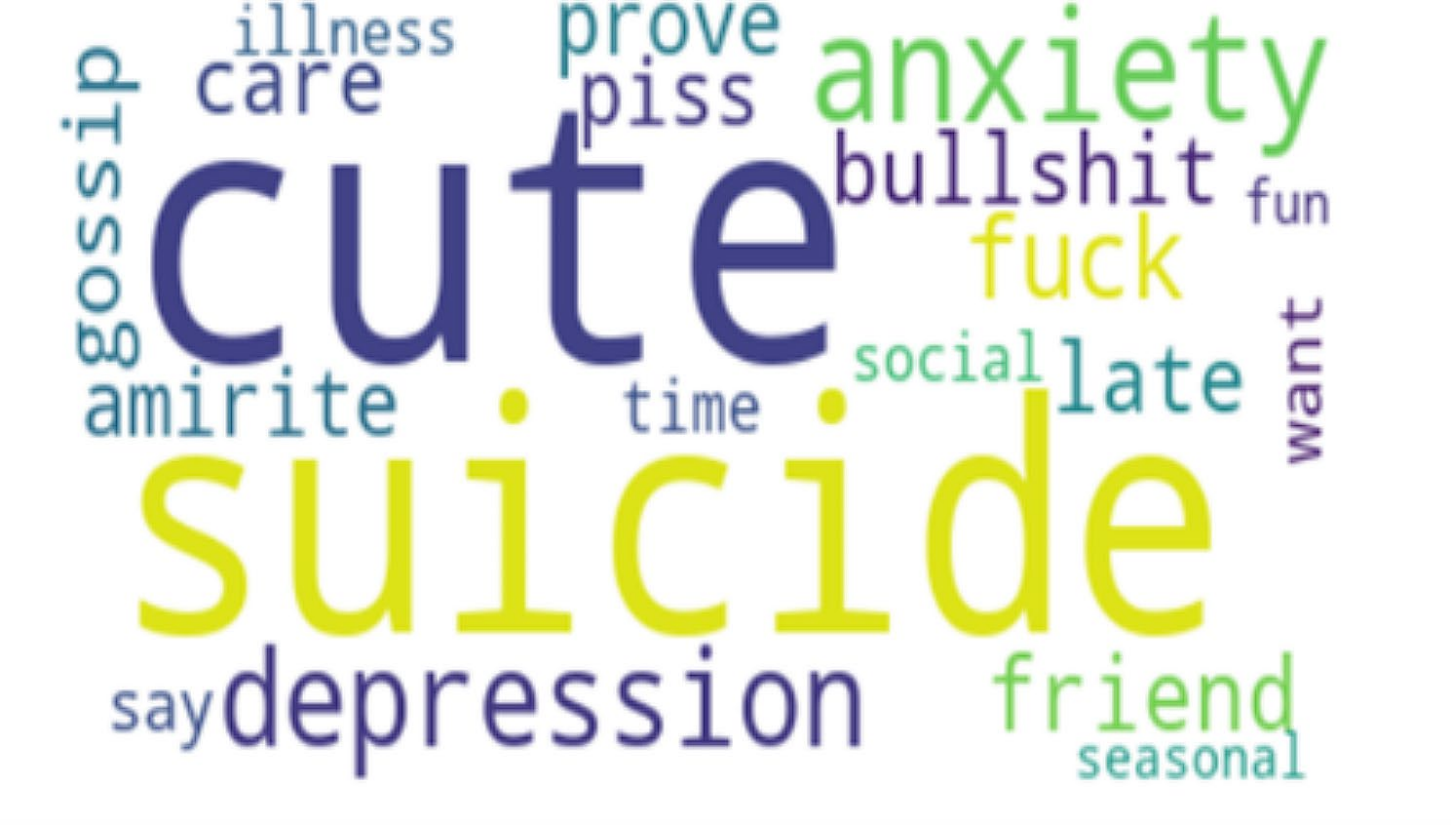
Word Cloud for Topic 1

**Fig. 2:**
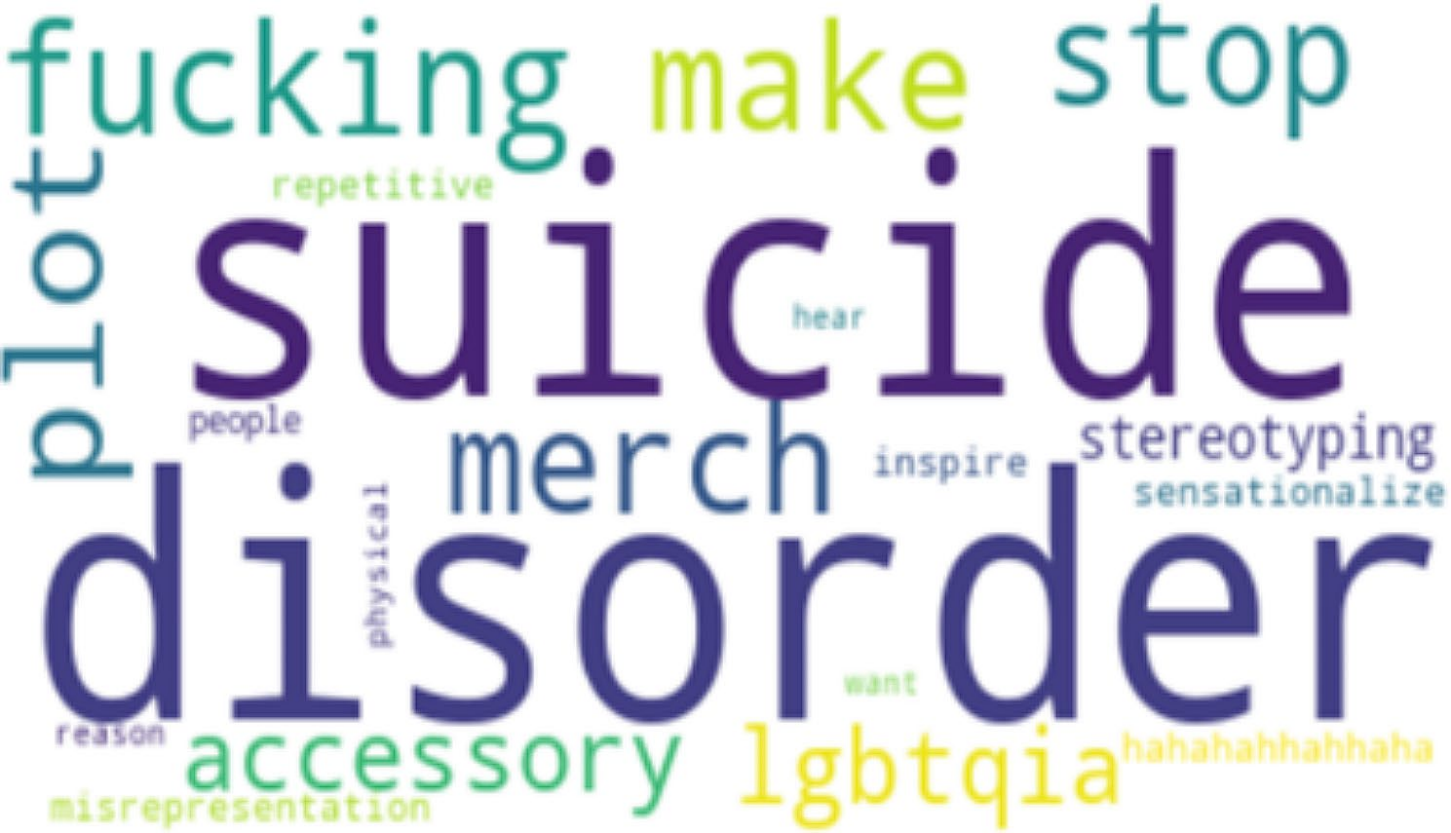
Word Cloud for Topic 2

**Fig. 3:**
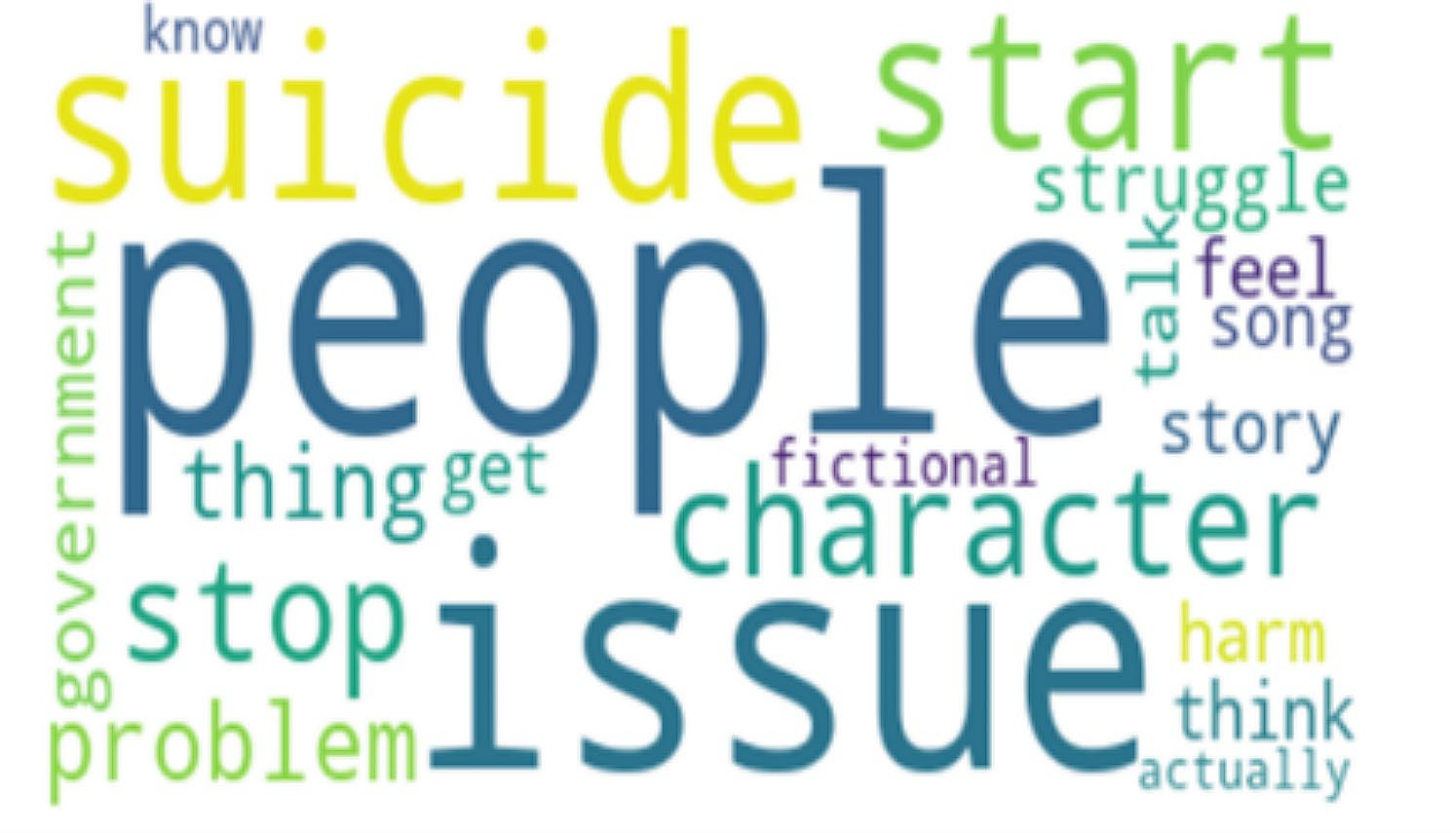
Word Cloud for Topic 3

**Fig. 4:**
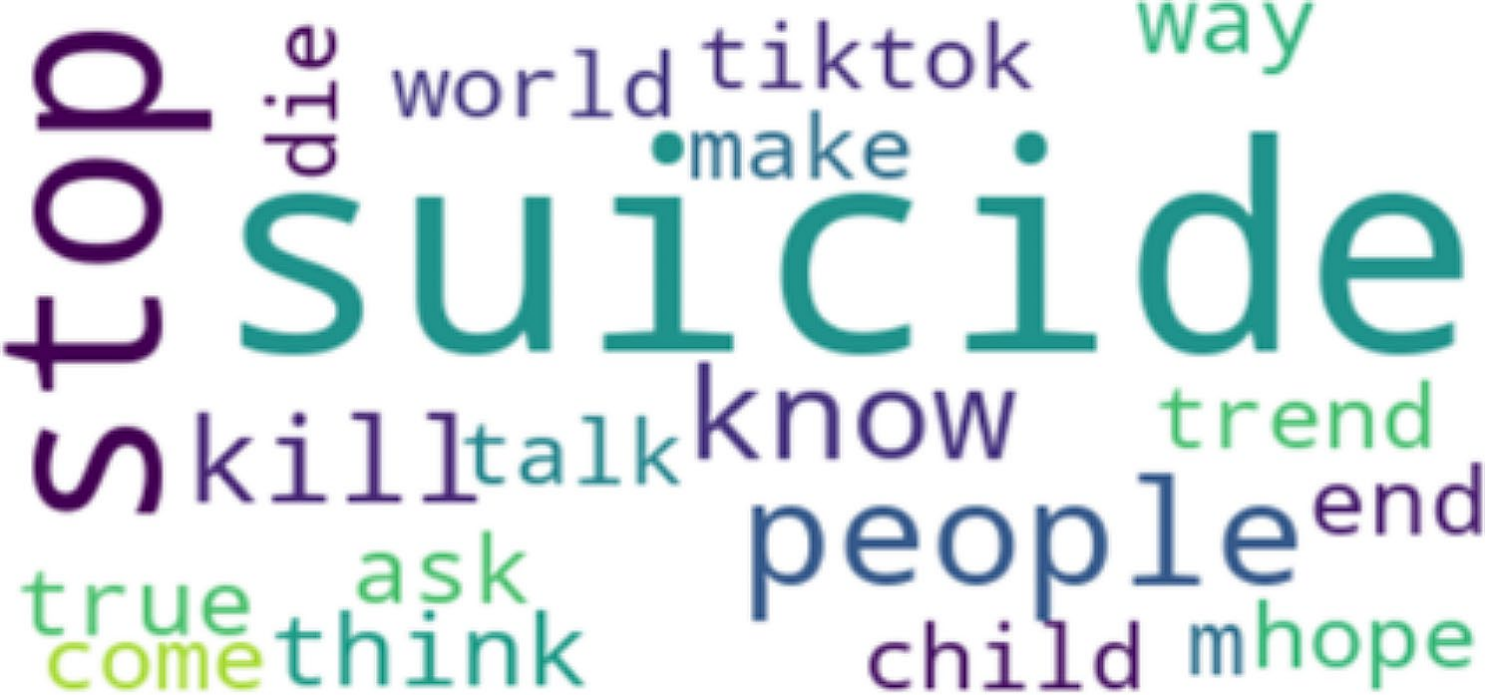
Word Cloud for Topic 4

**Fig. 5:**
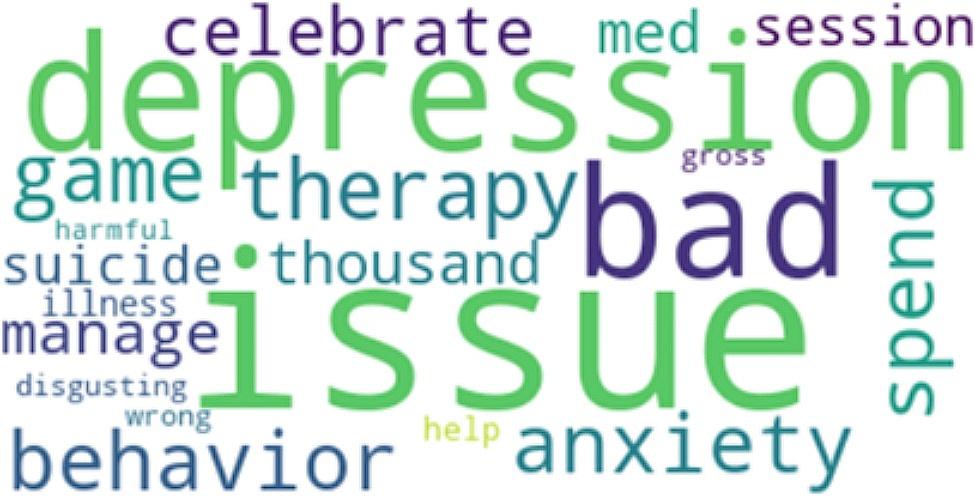
Word Cloud for Topic 5

To strengthen the credibility and provide context for the LDA model findings, which include the five topics in Table 2 the word frequencies in Table 3, and the visual representations of the word cloud in Figure 1–5, representing topics 1–5 respectively, we conducted a qualitative analysis to delve deeper into the underlying themes.

In Table 3 you will find data related to the top-ranked words and their frequencies based on the LDA analysis. This table indicates the top-ranked words identified by LDA analysis and the number of times each of these words appears in the dataset.

Consistent with prior research, we applied Braun and Clarke’s six-step thematic analysis: (i). familiarizing ourselves with the keyword data, (ii) creating initial codes, (iii) identifying themes, (iv) reviewing potential themes, (v) defining the themes, and (vi) reporting the results [53]. Our approach was iterative and reflective, allowing us to move back and forth through these phases.

To minimize biases, each of the team members independently went through this process and then cross-compared their findings. We reviewed all identified codes to ensure alignment with the identified topics. Six themes were categorized and named, aligning with the overarching meaning of the five identified topics. These six themes and their corresponding tweets can be found in Table 1.

**Table 1:** Themes and their corresponding tweets

Themes	Example topic	Tweets
1. Rejecting / Critiquing the glamorization of Mental Health	Cute Suicide Disorder	1. Anxiety is not cute at all for yall to make stupid tiktoks like that 2. I need some sort of petition to stop people from romanticizing mental health issues online cause this shit is actually living hell. It is not cute, fun, quirky, or unique. It makes living a regular life incredibly difficult and, in real life, will push people away from you. 3.Baby this depression is not cute or fun BRING ON THE MANIA SO I CAN FEEL ALIVE 4. When ur fat having depression is not cute 5. Depression is not cute at all, is it? It is a constant struggle to keep myself alive, folks. 6. One day I’ll get the help I need instead of romanticizing suicide on Twitter, not today, but one day 7. now why is #that group romanticizing suicide and murder an abusive father y’all need to stop romanticizing suicide and mental health issues on this app before i rek u 8. We need to stop romanticizing mental health disorders like adhd and autism and start monetizing them. 9. since when is being autistic somehow a trendy thing bc last I checked… it isn’t lol. i get called all sorts of god-awful things for being actually autistic and i think that romanticizing mental. 10.Need to get out of this bed and get cute cause this depression is not cute 11. Depression is not cute, and it is not a personality trait stop romanticizing it.
2. Monetization of Mental health by corporate organization	merch	1. They’s using repetitive plots, eh haha! Sensationalizing suicide and making a fucking merch and accessories out of it. 2. This is why businesses should know the background of what they’re selling, especially when it involves merchandise. You’re selling something you don’t know that you ended up unintentionally romanticizing suicide. It is honestly triggering. 3. Finally! I have always been bothered by merch with the phrase “to be one with the stars " like fls huhu stop just stop romanticizing suicide
3. Societal Misconception of Mental Health	People Issue	1. The amount of people romanticizing suicide in the comments is sickening. The firefighter saved someone’s life after they made an irrational decision. This is a real person with real family and friends, and a future ahead of them. Their life matters. 2. When and how cultural contagion happens is an interesting topic. The only analogy I have is about suicide. Talking about suicide does not increase rates of suicide (people think it does). But ROMANTICIZING suicide has caused contagion of the behavior. 3. More common than I think? I have read up on OCD last year A lot of you assume perfectionists have OCD which is very very wrong I have a friend with diagnosed OCD and I sure asl know how bad it is This era of youths and romanticizing mental health issues God abeg oh 4. Don’t romanticize your mental issues and romanticize working on yourself.
4. Role of Traditional Media and Social Media	Tiktok	1.tiktok is like if you breathe through your nose you have adhd, autism and bipolar. CAN we stop romanticizing mental health disorders? You have bpd because the clock app told you so? 2. It’s crazy how many TikTok’s I’ve seen of people romanticizing mental health issues…like girl it ain’t cute/trendy to have them… 3. “Between therapy becoming a trend and TikTok romanticizing mental health yall have all the language but none of the tools” 4. Yes they are. Social media has been romanticizing mental health issues of all sorts since I was in middle school. I’m in my 30s… 5.I’ve seen a few Yandere creators criticize people for romanticizing suicide in fiction, when they’re okay with **people** romanticizing MURDERS and abusive relationships in fiction? Like dude your making Yandere focused content, you really can’t be talking. 6. Romanticizing suicide I live in an area where the second most frequent cause of death for young **people** is suicide. This fact certainly got me thinking about the number of literary works I have taught which romanticize suicide.
5. Unfiltered realities of depression	Depression, Bad	1. My depression was bad I wouldn’t shower, brush my teeth or hair, clean my room nothing. I would legit work, come home, and sit on my computer all night and think about just driving till my car was empty and then killing myself Depression is not cute stop it. 2. I need to seek out fun again and not give a fuck if ppl get weird, butt hurt or jealous bc I want to have a good time. How long do I need to be a miserable adult for? Depression is not cute but I have to learn to push back before it consumes me. 3. I spend thousands on therapy and meds just to manage my depression and anxiety….Twitter they celebrate bad behavior by romanticizing mental health issues? 4. ppl love romanticizing mental health issues until someone shows the ‘bad’ symptoms
6. We are not romanticizing		1. I don’t think that I’m the only one! BUT again, it really doesn’t help things to call ppl “crazy” or “batshit” or say that they’re romanticizing suicide when they’re literally talking about a current event. ALSO, the suicide taboo is empirically bad at reducing suicides!! 2. I often vent about my depression and my suicidal thoughts and a few weeks ago, some chick literally said I was romanticizing suicide and was just whining for attention… Seriously, gfy!

Below, we will explain each theme and how they were expressed on Twitter. The corresponding tweets associated with each topic are shown in Table 1.

### Rejecting/critiquing the glamorization of mental health

Users’ responses conveyed strong critiques and concerns regarding the way mental health issues are portrayed on the platform. Many users expressed their vehement disapproval of the glamorization of mental health issues. They employed explicit language and terminology associated with specific mental health concerns to underscore their critique. Notably, one user remarked:

Anxiety is not cute at all for y’all to make stupid TikToks like that

Users also called attention to the glamorization of particular mental health conditions, highlighting their specific concerns. For instance:

Need to get out of this bed and get cute cause this depression is not cute” (Depression)

Within this theme, some users also expressed their strong desire to seek help and transform the way sensitive mental health topics are discussed on the platform. An illustrative example includes:

One day I’ll get the help I need instead of romanticizing suicide on Twitter, not today, but one day

Additionally, several users advocated for a fundamental shift in the portrayal and discourse surrounding mental health disorders. A notable tweet within this context is:

We need to stop romanticizing mental health disorders like adhd and autism

These tweets collectively shed light on users’ critiques and expressions within Theme 1.

### Monetization of mental health by corporate organization/confronting the commercialization of mental health (CMH)

Twitter users expressed their concerns and critiques regarding the commercialization of mental health issues through merchandise. In response to the repetitive use of certain themes, one user provided a critique:

They’re using repetitive plots, eh haha! sensationalizing and romanticizing suicide and making a fucking merch and accessories out of it.

Some users also highlighted the significance of businesses having a comprehensive understanding of what they are selling, particularly when it involves merchandise related to mental health:

This is why businesses should know the background of what they’re selling, especially when it involves merchandise. You’re selling something you don’t know that you ended up unintentionally romanticizing suicide. It is honestly triggering.

Users also expressed their concerns regarding specific phrases used on merchandise items:

Finally! I have always been bothered by merch with the phrase ‘to be one with the stars’ like fls huhu stop just stop romanticizing suicide.

These tweets collectively reflect users’ concerns about how phrases used on merchandise sold for profit can intentionally or unintentionally promote romanticization of mental health.

### Societal misconception of mental health

Within the theme of “Societal Misconception of Mental Health,” users shared their perspectives on common societal misunderstandings and misconceptions surrounding mental health. Many users expressed their concerns about the societal misconception of romanticizing suicide and its harmful consequences:

The amount of people romanticizing suicide in the comments is sickening. The firefighter saved someone’s life after they made an irrational decision. This is a real person with real family and friends, and a future ahead of them. Their life matters.

When and how cultural contagion happens is an interesting topic. The only analogy I have is about suicide. Talking about suicide does not increase rates of suicide (people think it does). But ROMANTICIZING suicide has caused contagion of the behavior.

Some users took the opportunity to challenge common misconceptions about mental health, aiming to correct these misunderstandings:

More common than I think? I have read up on OCD last year. A lot of you assume perfectionists have OCD, which is very, very wrong. I have a friend with diagnosed OCD, and I sure asl know how bad it is.

Users also criticized the societal misconception of romanticizing mental health issues, particularly among young people:

This era of youths and romanticizing mental health issues. God abeg oh.

Finally, users encouraged a more balanced perspective on mental health, emphasizing the importance of self-improvement rather than romanticizing mental health issues:

Don’t romanticize your mental issues and romanticize working on yourself.

### Role of Traditional Media and Social Media

​​ Users voiced their observations and concerns regarding the portrayal of mental health on these platforms. Many users denounced the tendency to romanticize mental health disorders on platforms like TikTok, emphasizing the need to address this issue:

TikTok is like if you breathe through your nose you have adhd, autism and bipolar. CAN we stop romanticizing mental health disorders? You have bpd because the clock app told you so?

It’s crazy how many TikTok’s I’ve seen of people romanticizing mental health issues…like girl it ain’t cute/trendy to have them…

Between therapy becoming a trend and TikTok romanticizing mental health y’all have all the language but none of the tools.

Some users observed that the romanticization of mental health issues on social media has been a long-term trend:

Yes they are. Social media has been romanticizing mental health issues of all sorts since I was in middle school. I’m in my 30s…

Additionally, a user pointed out inconsistencies in media criticism related to the romanticization of mental health:

I’ve seen a few Yandere creators criticize people for romanticizing suicide in fiction when they’re okay with people romanticizing MURDERS and abusive relationships in fiction? Like dude you’re making Yandere focused content, you really can’t be talking.

### Unfiltered realities of depression

Within the theme of “Unfiltered Realities of Depression,” users candidly shared their personal experiences and unfiltered perspectives on living with depression.

Some users provided raw and unfiltered insights into their personal experiences with depression:

My depression was bad. I wouldn’t shower, brush my teeth or hair, clean my room, nothing. I would legit work, come home, and sit on my computer all night and think about just driving till my car was empty and then killing myself.

Others expressed a strong desire to break free from the grip of depression and regain a sense of joy in their lives:

I need to seek out fun again and not give a fuck if ppl get weird, butt hurt or jealous bc I want to have a good time. How long do I need to be a miserable adult for?

Depression is not cute but I have to learn to push back before it consumes me.

Users also critiqued the way Twitter seems to celebrate bad behavior while romanticizing mental health issues:

I spend thousands on therapy and meds just to manage my depression and anxiety….Twitter they celebrate bad behavior by romanticizing mental health issues? Baket?

Another user pointed out the inconsistencies in how mental health issues are perceived, especially when the “bad” symptoms are revealed:

Ppl love romanticizing mental health issues until someone shows the ‘bad’ symptoms.

These tweets collectively reflect users’ unfiltered experiences and perspectives within the theme of “Unfiltered Realities of Depression,” emphasizing the harsh realities of living with depression.

### We are not romanticizing

Within the theme of “We are not romanticizing,” users defended themselves and others against accusations of romanticizing suicide or mental health struggles. Many users took a stand against being labeled as romanticizing suicide or mental health issues and emphasized that discussing these topics is crucial:

I don’t think that I’m the only one! BUT again, it really doesn’t help things to call ppl ‘crazy’ or ‘batshit’ or say that they’re romanticizing suicide when they’re literally talking about a current event.

ALSO, the suicide taboo is empirically bad at reducing suicides!!

### Some users also shared their frustration with being accused of romanticizing suicide or seeking attention”

I often vent about my depression and my suicidal thoughts and a few weeks ago, some chick literally said I was romanticizing suicide and was just whining for attention… Seriously, gfy!

These tweets collectively reflect users’ strong reactions and defenses within the theme of “We are not romanticizing

## Discussion

With romanticization of mental health issues, discourses on Twitter are complicating the stigmatization and destigmatization dichotomy of mental health issues. The theme of rejecting the glamorization of mental health in the tweets analyzed is indicative of the findings from several studies that have considered the negative effects of glamorizing mental health on social media [12, 54]. Similar to previous studies, society is now moving mental illness a step further from being stigmatized to glorified [21]. Such a shift masks the attention that needs to be paid to mental illness. The harmful effect of this trend of glamorization of health issues has been emphasized by researchers, medical (psychiatry) experts, and institutions [1, 55]. Unique to our study, we highlight the outcry for people in their tweet to end this glorification act. Interestingly, Twitter users are calling for legal actions to be taken against this act because it is not “cute”. People believed that engaging in these glamorization acts shift attention from the “actual” or “real” symptoms that people experiencing mental illness have to deal with such as depression anxiety, personality disorder, and schizophrenia. Embedded in this call is the misformation and disformation that is spread within these glamorized tweets on mental illness.

In addition, our findings on monetization of mental health by corporate organizations/confronting the commercialization of mental health (CMH) explains the frustration Twitter users expressed towards businesses’ inhumane acts of promoting romanticization of mental illness through their advertisement, brands, logo, slogans or even products. Businesses over the years have been blamed for their insatiable drive to make money even out of most illnesses including mental illness. Some influencers and celebrities used this opportunity to increase their followers or likes/comments whereas others created various forms of clubs. In a story published on invisible illness medium, several bloggers explored some of these businesses created and how they are making money out of mental health. For instance, Corinna Kopf’s anxiety merch developed through a clothing line were criticized as well as appreciated by others. This suggests businesses are co-opting mental health issues into trends and fashion statements to gain profit. Given that research has also shown how people are influenced mostly by influencers or celebrities in several ways [56] commercializing mental health issues through the use of symbols and phrases in sale of merchandise for profit downplays the severity of mental illness particularly in relation to suicide. From our findings, Twitter users emphasized in their discussion the societal misconceptions of mental health that are propagated and the need for these to be corrected. Data from the Substance Abuse and Mental Health Services Administration (SAMHSA) explained several myths that have been associated with people living with mental conditions, which the participants in this study confirmed. Due to these misconceptions, people are deprived of opportunities. Yu et al. [28] confirmed the lack of knowledge exhibited about depression on social media ranging from quality of life, access to good jobs, social networks, healthcare satisfaction and general care from people [5].

Being a stereotype group, Twitter users projected their dismay about the misinformation/disinformation and lack of knowledge that is out there about mental health. These myths and misconception have been disclosed to consequently reinforce the already existing discrimination public and self-stigma that people with mental conditions are faced with [57–59]. Romanticizing mental illness on social media has worsened these effects and caused people to engage in self harm, wrong diagnosis, and death.

We found that the role of traditional and social media is pivotal in shaping public understanding of mental health. The influence of media platforms, both traditional and social, in shaping public understanding of mental health has become a subject of growing concern [60, 61]. This is because they play a very important role in framing the discussion on mental health [62]. Certain media platforms have played a constructive role in addressing mental health concerns by disseminating messages aimed at educating and enlightening individuals about mental well-being, as well as promoting the importance of seeking support [63]. Several researchers argue that some platforms have failed to fulfill their responsibility in educating and enlightening the public about mental health, instead, potentially glamorizing, and idealizing it [9, 11, 64].

In our findings, participants expressed apprehensions regarding potential glorification and oversimplification of mental health issues on social media e.g. Tik Tok and other traditional platforms. These findings align with the observation made by Jadayel et al. [1] that young individuals tend to romanticize mental health disorders on social media platforms. This raises pertinent questions about the ethical responsibility of media platforms in portraying mental health accurately and responsibly. This may give credence to Frost and Casey’s [65] argument that people seeking online support might be susceptible to increased levels of exposure to suicidal ideation, which ratifies the need for online posting monitoring.

On the contrary, other findings example tweets like:

My depression was bad I wouldn’t shower, brush my teeth or hair, clean my room nothing. I would legit work, come home, and sit on my computer all night and think about just driving till my car was empty and then killing my self Depression is not cute stop it.,

portrays Twitter as a significant platform for individuals to share unfiltered, real-life experiences of grappling with mental health, particularly depression. These narratives shed light on the raw and often challenging aspects of living with this condition. In doing so, they provide a counterbalance to potentially romanticized portrayals, emphasizing the importance of authenticity in mental health discussions. This finding aligns with research by Berry et al. [66] that found that Twitter provides an online community that allows communication on mental health to flourish, awareness to be raised and space for authentic mental health related discussions and self- disclosure. Moreover, the narratives draw attention to practical challenges faced by individuals, such as the financial burden of accessing therapy. This aspect underscores the urgent need for increased accessibility to mental health resources, ensuring that individuals have the support they require. Although these may be seen as romanticizing mental health, it might be important for policy makers and public health agencies to use as a feedback platform to gain valuable feedback from members of the society having mental health challenges.

Along the same line, our final finding “We are not romanticizing” indicates how some users vehemently reject the tag on them that portrayed them as glorifying mental health. Users passionately advocate for authentic, unvarnished discussions surrounding mental health, while vehemently asserting that their intentions are far from romanticizing the very real struggles individuals face. Nevertheless, this outcome contradicts the conclusions drawn by Chan and Sireling [67], who argue that the recent positive media coverage and romanticization of mental health challenges of bipolar disorder might contribute to its growing popularity as a self-diagnosed condition.

In all these contradictions, an important aspect of these findings is the active challenge against societal taboos surrounding discussions of suicide. Users courageously step into a realm that has long been shrouded in silence and stigma. By doing so, they contribute to a broader movement that seeks to deconstruct harmful narratives and replace them with open, empathetic conversations. The importance of this cannot be overstated, as these conversations are instrumental in reducing the isolation felt by individuals struggling with suicidal thoughts. They pave the way for increased understanding, support, and access to resources for those who need it the most. Additionally, this finding also underscores a fundamental human right: the right to vent about mental health challenges without facing stigmatization as a romanticizer. This reaffirms the validity of individual experiences and emphasizes that venting is not an endorsement of romanticization, but a crucial aspect of processing and coping [66]. By upholding this right, users foster an environment where other individuals can express themselves without fear, and where their experiences are acknowledged and respected.

## Conclusion

In conclusion, this study explored the complex terrain of Twitter conversations about mental health, uncovering a range of issues and viewpoints related to the phenomena of romanticizing mental health. We discovered six major themes through content analysis and topic modeling: we are not romanticizing; we reject/critique the glamorization of mental health; corporate organizations monetize of mental health; societal misconceptions about mental health; the role of traditional media and social media; and the glamorization of mental health.

Our research highlighted how critical it is to address the complicated problem of social media romanticization of mental health. Users voiced their worries, criticized the glamorization of mental health issues, and demanded more appropriate representations of diseases associated with mental health. These Twitter conversations show how the conversation surrounding mental health is changing, moving from stigmatization to a growing concern about the romanticization of mental illness.

### Research and practical implications

This study significantly contributes to the growing body of research on mental health discussions in the digital era. Our study underscores the importance of conducting platform-specific analyses to understand how users engage in discussions about sensitive topics like mental health. Our study calls for further research on the romanticization of mental health in the context of social media. Future studies can explore how these conversations have changed over time, considering how different events and trends may impact the conversation. Additionally, examining other social media platforms and their unique dynamics is essential for a comprehensive understanding of this issue.

Understanding the motivations and demographics of users engaging in these discussions can provide valuable insights. Research could delve deeper into user intentions, whether they aim to raise awareness, seek support, or engage in performative behavior. Traditional and social media platforms must recognize their role in shaping mental health perceptions responsibly. Mental health organizations and advocates can leverage Twitter as a platform to engage with users actively discussing these issues. By providing accurate information and support, mental health experts can contribute to a more informed and empathetic online community.

Promoting digital literacy and responsible online behavior is crucial. Users should be educated about the potential harms of glamorizing mental health and encouraged to engage in meaningful, empathetic conversations. Online support groups and communities can benefit from our findings by emphasizing the importance of authenticity. Encouraging members to share unfiltered experiences can foster a sense of belonging and understanding.

### Limitations

The study primarily relied on data from Twitter, offering a diverse dataset, but it’s important to acknowledge its limitations. Firstly, the dataset collected on Twitter, while informative, may not encompass the entirety of discussions regarding the romanticization of mental health, potentially overlooking conversations occurring on other social media platforms or in private settings.

Additionally, the study’s use of specific keywords for data collection could have unintentionally excluded relevant discussions that didn’t explicitly employ those keywords, potentially leading to the omission of valuable insights. While the findings provide valuable insights into discussions among Twitter users, they may not be entirely representative of the broader Twitter user population. Additionally, it’s important to note that the dataset collected from Twitter, although informative, was not extensive in size and was limited to a relatively short time frame. This temporal constraint may have restricted the study’s ability to capture the full spectrum of discussions and developments related to the romanticization of mental health on the platform over a more extended period.

Furthermore, due to privacy concerns and the public nature of Twitter data, the study was unable to access detailed demographic information about users, which could have offered valuable contextual information to enhance the analysis. Additionally, while the study employed topic modeling to identify key themes, it’s essential to acknowledge that this approach might not capture the full depth of user intentions and emotions in their discussions.

Future studies should take these limitations into account in order to offer a broader understanding of the phenomenon.

Table 1 above displays the six themes that were manually categorized and named based on the identified topics from the topic modeling. It also highlights words related to each theme, as well as corresponding tweets specific to each theme.

Table 2 above represents the five main topics from the LDA topic modeling of our Twitter dataset, including the top ten words for each topic and example tweets that support the findings.

**Table 2:** Five Topic Models

Topic	Top 10 Words	Example
Topic 1	“suicide” “cute” “depression” “anxiety” “bullshit” “piss” “care” “prove” “gossip” “amirite”	“I often vent about my depression and my suicidal thoughts and a few days ago, chick literally said I was romanticizing suicide and was just whining for attention… Seriously!, gfy!”
Topic 2	“suicide” “disorder” “merch” “stereotyping” “plot” “fucking” “accessory” “make” “stop” “misrepresentation”	“This is why businesses should know the background of what they’re selling, especially when it involves merchandise. You’re selling something you don’t know that you ended up unintentionally romanticizing suicide. It is honestly triggering.”
Topic 3	“people” “suicide” “start” “issue” “character” “stop” “thing” “struggle” “think” “problem”	“The amount of people romanticizing suicide in the comments is sickening. The firefighter saved someone’s life after they made an irrational decision. This is a real person with real family and friends, and a future ahead of them. Their life matters.”
Topic 4	“suicide” “stop” “kill” “know” “people” “world” “tiktok” “die” “trend” “think”	“One day I’ll get the help I need instead of romanticizing suicide on Twitter, not today but one day”
Topic 5	“depression” “issue” “therapy” “bad” “thousand” “celebrate” “game” “anxiety” “behavior” “spend”	“Depression is not cute at all, is it? It is a constant struggle to keep myself alive, folks.”

**Table 3:** Frequencies of the top ranked words

Rank	Words	Frequency	Rank	Words	Frequency
1	suicide	325	13	med	43
2	issue	99	13	session	43
3	anxiety	98	14	fucking	41
4	depression	94	15	fuck	39
5	people	92	15	plot	39
6	cute	80	16	merch	38
7	make	52	17	stereotyping	37
8	bad	50	17	hahahaha	37
9	therapy	48	17	misrepresentation	37
10	spend	47	17	sensationalyze	37
11	behavior	45	17	repetitive	37
12	game	44	17	inspire	37
13	thousand	43	17	lgbqia	37
13	manage	43	18	accessory	36
13	celebrate	43	19	ate	19

## Data Availability

The datasets generated and/or analyzed will be made available from the corresponding author upon request.
